# Estimating the clinical benefits of vaccinating boys and girls against HPV-related diseases in Europe

**DOI:** 10.1186/1471-2407-13-10

**Published:** 2013-01-08

**Authors:** Rémi Marty, Stéphane Roze, Xavier Bresse, Nathalie Largeron, Jayne Smith-Palmer

**Affiliations:** 1 HEVA, 186 Avenue Thiers, Lyon, 69006, France; 2 SPMSD SNC, Lyon, France; 3 Ossian Health Economics and Communications, Basel, Switzerland

**Keywords:** Human papillomavirus, Vaccination, HPV-related cancer, Genital warts, HPV-related disease, Cervical cancer

## Abstract

**Background:**

HPV is related to a number of cancer types, causing a considerable burden in both genders in Europe. Female vaccination programs can substantially reduce the incidence of HPV-related diseases in women and, to some extent, men through herd immunity. The objective was to estimate the incremental benefit of vaccinating boys and girls using the quadrivalent HPV vaccine in Europe versus girls-only vaccination. Incremental benefits in terms of reduction in the incidence of HPV 6, 11, 16 and 18-related diseases (including cervical, vaginal, vulvar, anal, penile, and head and neck carcinomas and genital warts) were assessed.

**Methods:**

The analysis was performed using a model constructed in Microsoft®Excel, based on a previously-published dynamic transmission model of HPV vaccination and published European epidemiological data on incidence of HPV-related diseases. The incremental benefits of vaccinating 12-year old girls and boys versus girls-only vaccination was assessed (70% vaccine coverage were assumed for both). Sensitivity analyses around vaccine coverage and duration of protection were performed.

**Results:**

Compared with screening alone, girls-only vaccination led to 84% reduction in HPV 16/18-related carcinomas in females and a 61% reduction in males. Vaccination of girls and boys led to a 90% reduction in HPV 16/18-related carcinomas in females and 86% reduction in males versus screening alone. Relative to a girls-only program, vaccination of girls and boys led to a reduction in female and male HPV-related carcinomas of 40% and 65%, respectively and a reduction in the incidence of HPV 6/11-related genital warts of 58% for females and 71% for males versus girls-only vaccination.

**Conclusions:**

In Europe, the vaccination of 12-year old boys and girls against HPV 6, 11, 16 and 18 would be associated with substantial additional clinical benefits in terms of reduced incidence of HPV-related genital warts and carcinomas versus girls-only vaccination. The incremental benefits of adding boys vaccination are highly dependent on coverage in girls. Therefore, further analyses should be performed taking into account the country-specific situation. In addition to clinical benefits, substantial economic benefits are also anticipated and warrant further investigation as do the social and ethical implications of including boys in vaccination programs.

## Background

The human papillomavirus (HPV), in particular subtypes 6, 11, 16 and 18 are responsible for a number of conditions including genital warts, recurrent respiratory papillomatosis, a subset of head and neck cancers as well as vaginal, vulvar, cervical and anal cancers in females and penile and anal cancers in males. There is a wealth of published literature relating to reductions in the clinical and economic burden of cervical cancer, due in part to the success of pap screening programs and the introduction of the HPV vaccines. This has meant that the burden of other HPV-related cancers, particularly those occurring in males, is often overshadowed and as such is less well characterized [1]. In particular, data from a recent meta-analysis by De Vuyst *et al*. report that 40.4% of vulvar carcinomas, 69.9% of vaginal carcinomas and 84.3% of anal carcinomas are due to HPV (all subtypes) [2]. Additionally, around 22% of head and neck cancers test positive for HPV [3] and around 50% of penile carcinomas are specifically due to HPV 16 or 18 [4]. Moreover, it is estimated that approximately 30% of all HPV-related cancers occur in males, which in European males corresponds to approximately 17,000 cancer cases per year, of which over 15,000 are attributable to HPV 16.

The burden of HPV-related non-cervical cancers is, in many settings, comparable to or greater than that associated with cervical cancer. For example, in France the total cost (2006/2007 EUR) of HPV-related cancers was EUR 240 million, of which only EUR 84 million was attributable to invasive cervical cancer [5]. Furthermore, whilst the clinical and economic burden associated with cervical cancer has declined notably in recent years, and is likely to decline even more in the coming decades owing to the introduction of the bivalent and quadrivalent HPV vaccines, the situation in relation to other HPV-related cancers is less well characterized. Epidemiological data from the UK has suggested that the age-standardized incidence of vulvar and vaginal cancer in females and penile cancer in males has remained relatively unchanged since the 1960s, but that the incidence of anal cancer has increased substantially in both males and females over the same time period [6]. Additionally, a number of European studies have shown that the incidence of HPV-related head and neck cancers in men has been increasing in recent decades [7,8].

In addition to HPV-related cancer, HPV 6 and 11 are responsible for 90% of cases of genital warts, which are in turn responsible for an estimated 9–10% of all visits to sexual health clinics [9,10]. Moreover, analysis of temporal trends in a number of settings has shown that the incidence of new cases of genital warts has increased substantially in the past few decades, [11] such that each year in Europe an estimated 287,000 to 326,000 cases of HPV 6/11-related genital warts are reported in males [1]. In terms of economic burden, a US based study by Hoy *et al*. reported that in 2004 the direct costs of genital warts were USD 104 million for females and USD 119 million for males [12]. Similarly, in France the estimated treatment cost per episode (direct medical costs, societal perspective) of genital warts is EUR 483 (2005 EUR), leading to a total annual burden of EUR 23 million, which is just under half of the total annual management costs associated with cervical cancer in France [13]. Genital warts also have a negative impact on quality of life and are associated with indirect costs with Castellsague *et al*. reporting that 16.7% of patients had used using sick leave due to genital warts [14,15].

Two vaccines exist that provide protection against HPV-related diseases, a bivalent vaccine that provides protection against HPV 16 and 18 and a quadrivalent vaccine that provides protection against HPV 6, 11, 16 and 18. Although HPV vaccination was first approved for use in females, the quadrivalent vaccine has subsequently demonstrated efficacy in terms of preventing HPV-related disease in males and is approved and recommended for use in males in both the US and Australia for the prevention of anal cancer, anal intraepithelial neoplasia and genital warts. In a 2011 study in over 4,000 males aged 16–26 years, the efficacy of the quadrivalent vaccine against HPV 6, 11, 16 or 18-related external genital lesions was 92.4% among heterosexual men and 79.0% among men who had sex with men. Moreover, no HPV 16/18-related lesions were reported in the vaccine group (a total of 3 HPV 16/18-related lesions were reported in the placebo group) [16].

A large number of cost-effectiveness analyses conducted in a number of different settings have shown that vaccination of females is cost-effective in comparison with no vaccination; however, there are relatively few data relating to the incremental benefits of vaccinating both males and females in comparison with female only vaccination programs. The consensus among the few studies that have been conducted is that gender-neutral vaccination programs are likely to further reduce the incidence of HPV-related disease in both males and females [17,18]. For example, the findings of Brisson *et al*. indicated that a gender-neutral vaccination program (vaccinating 12-year old boys and girls) would result in an incremental reduction in the incidence of HPV 16/18 infection of 16% in females and 23% in males versus female only vaccination over a 70 year-long period (assuming a 99% vaccine efficacy, 20-year long duration of protection and 70% vaccine coverage) [19].

On a population level, the effectiveness of vaccination programs has been shown to be dependent on uptake rates and national strategies in relation to vaccination vary between settings. Organized school-based vaccination programs have a very high uptake in the target population, although only 13% of young women in the European Union are covered by such programs. In settings where vaccination is provided on demand (e.g. Germany and France) vaccination rates are approximately 50% [20]. Introducing policies to increase vaccine uptake rate among girls would likely lead to a greater reduction in the incidence of HPV-related disease. Alternatively, vaccinating both boys and girls could also lead to reduced incidence of HPV-related disease amongst both males and females. A recent analysis by Bogaards *et al*. suggested that increasing vaccine coverage among girls was a more effective strategy in terms of reducing overall rates of HPV infection than vaccinating boys [21]. As such, in the current exploratory analysis the long-term clinical impact of vaccinating both boys and girls is investigated in the European setting. In particular, the impact of girls-only versus girls and boys vaccination on the incidence of male HPV-related disease (anal, penile and head and neck carcinoma and genital warts) is investigated. As screening and vaccination policies as well as uptake rates vary across Europe it should be noted that the current analysis provides a mean estimate only across Europe and country-specific analyses are required for more accurate estimates of the incremental benefits of vaccination of both girls and boys against HPV 6, 11, 16 and 18.

## Methods

### Model structure

Epidemiological estimates for HPV-related disease were based mainly on a previously published dynamic transmission model (a detailed description of which is provided by Elbasha *et al*. [22] and Dasbach *et al*. [23]). In summary, Elbasha *et al*. constructed a population dynamic model to account for both the direct and indirect effects of vaccination. Within the model, the population is divided into groups based on age and gender, which allows the patterns of HPV transmission among sexually active groups to be modeled accurately. Structurally, the model can be considered as containing three key components: HPV transmission, cervical cancer development and the occurrence of genital warts. Our analysis was performed based on a two-stage calculation. As a first stage, the US-based dynamic transmission model of Elbasha *et al*. was run for each vaccination strategy assessed (in this instance 12-year old girls-only vaccination program and a 12-year old girls and boys vaccination program). A screening only scenario was also run, which provided a common baseline comparator. The dynamic transmission model outputs absolute incidence of HPV-related disease cases per year and is run over a 100-year time span for each of the two vaccination strategies.

In the second stage, the annual proportional reductions in disease incidence due to a given vaccination strategy versus baseline scenario (screening only) were derived for each HPV-related disease within Microsoft Excel 2003. These proportional reductions were then applied to European incidence data reflecting incidences prior to HPV vaccination implementation. The present analysis is then able to derive avoided outcomes (i.e. cases of HPV-related diseases avoided) versus screening only for both the 12-year old girls-only vaccination program and the 12-year old girls and boys (gender neutral) vaccination program. The difference between the girls-only and gender-neutral vaccination is also presented. Internal validation of this two-step procedure was achieved by being able to replicate US and UK results from Dasbach *et al*. 2008 [23] and Elbasha *et al*. 2010 [17].

### Model input data

The analysis incorporated female-specific conditions including HPV 6/11/16/18 related cervical, vulvar and vaginal intraepithelial neoplasia states and carcinoma, penile intraepithelial neoplasia and carcinoma in males and genital warts, anal intraepithelial neoplasia and carcinoma, and head and neck cancers in both males and females.

Epidemiological input data relating to the incidence of HPV-related disease in Europe were derived from previously published epidemiologic studies by Bonnani *et al*. [20] and Hartwig *et al*. [1] (Table 1). In line with these sources, the definition of Europe within our analysis encompasses a total of twenty six countries including all European Union countries (except Greece, Hungary, Luxemburg, and Romania) as well as three countries (Iceland, Norway and Switzerland) outside the European Union.

**Table 1:** **Epidemiological input data used in the model**^**a**^

**Gender**	**Cancer sites (ICD 10 code)**	**Expected number of new cases, irrespective of HPV status**	**HPV prevalence by site (%)**	**Expected number of new cases attributable to HPV**	**Prevalence of HPV 16/18 in HPV-positive cancers (%)**	**Expected number of new cancer cases attributable to HPV 16/18**	**Prevalence of HPV 6/11 in HPV-positive warts (%)**	**Expected number of new cancer cases attributable to HPV 6/11**
Male	Head and neck^b^	67,354		14,098		12,707		
	Anus (C21)	2,162	84.2	1,821	87.1/6.2	1,699		
	Penis (C60)	3,178	46.7	1,484	60.2/13.4	1,091		
	Genital warts	380,961					85.5	325,722
Female	Cervical cancer	30,517	-	-	59.2/17.0	23,254		
	Vaginal	1,869	69.9	1,306	76.8/10.9	1,146		
	Vulvar	7,384	40.4	2,983	79.7/10.9	2,702		
	Anus (C21)	3,727	84.3	3,141	87.1/6.2	2,929		
	Head and neck	13,448		2,715		2,531		
	Genital warts	337,963					85.5	288,959

HPV, human papillomavirus.

^a^In total 26 countries were considered in the analysis for incidence estimates, i.e. all European Union countries (except Greece, Hungary, Luxembourg, and Romania). Three countries outside the European Union were included (Iceland, Norway and Switzerland).

^b^includes several ICD 10 codes related sites (i.e., tongue, gum of the mouth, floor of the mouth, palate, tonsil, piriform sinus), hypopharynx and larynx sites.

Vaccine efficacy for transient and persistent infections and compliance input data used were derived from a previously published model [17] and are described in Table 1 and Table 2.

**Table 2:** Vaccine efficacy parameters and assumptions

**Gender**	**Male**				**Female**			
**HPV genotype**	**6**	**11**	**16**	**18**	**6**	**11**	**16**	**18**
**Against transient infection**^**†,‡**^								
- Cervical, vaginal & vulvar diseases	―	―	41.1	62.1	―	―	76.0	96.3
- Genital warts & HPV 6, 11	49.0	57.0	―	―	76.1	76.1	―	―
**Against persistent infection**								
- Anal disease	―	―	78.7	96.0	―	―	98.8	98.4
- Cervical, vaginal & vulvar diseases	―	―	―	―	―	―	98.8	98.4
- Penile disease	―	―	78.7	96.0	―	―	―	―
**Against individual diseases**								
- Genital warts	84.3	90.9	―	―	98.9	100.0	―	―

Unit: percentage. Values were derived from [17].

^†^Efficacy against genital infection in males is assumed to prevent transmission of genital infection to females, and vice versa.

^‡^Efficacy for 1 and 2 doses assumed to be 23% and 45% of efficacy of the full 3 doses, respectively.

*Efficacy against anal, head and neck, penile cancers is conferred through protection against infection only.

### Assumptions

The vaccine (both for the girls-only and boys and girls vaccination programs) was assumed to be administered to 12-year olds. A number of assumptions were made with regard to the vaccine coverage, compliance and duration of vaccine protection (Table 3). A vaccine coverage of 70% was assumed for girls in the girls-only vaccination program and for both genders in the gender-neutral vaccination program. Both vaccination program strategies were assumed to achieve 70% coverage rate starting from the first year of implementation (no transition period was assumed). This figure represents the proportion of either girls or boys that received at least one vaccination dose out of the full three doses vaccination course. Imperfect adherence to the scheduled vaccination course was also taken into account in line with the previously published analysis of Elbasha and Dasbach (Table 3) [17]. Decreased vaccine efficacy was also assumed for those having received either one or two doses in comparison with those who were fully vaccinated (three doses) (Table 2). In base case, duration of vaccine protection was that of patient lifetimes.

**Table 3:** Base-case input parameters used in the model

**Parameter**	**Values**
**Vaccine uptake, both sexes**	
Cumulative percentage of vaccine uptake (first dose) among 12-year olds	70%
**Vaccine adherence (probability of second and third dose), both sexes**	
Percentage of individuals receiving the second dose given first dose	79.7%
Percentage of individuals receiving the third dose given second dose	63.5%
**Duration of protection (years)**	lifelong

HPV, human papillomavirus. Values were derived from [17].

Apart from vaccination-related parameters, all US and disease-specific parameters related to underlying demographic US population (pyramidal structure of age), HPV transmission and progression to disease, cervical and vaginal screening programs were assumed to be applicable to European settings [17].

### External validation

The use of US-based input parameters for application in the European setting seems reasonable when comparing vaccination impact at different points in time, in terms of cervical cancer incidence, published either with the US base case model (Elbasha *et al*. 2007 [22]) or its adaptation for the UK setting (Dasbach *et al*. 2008 [23]). Components of the model that were modified for the UK included the demographic characteristics (e.g. mortality), screening, and treatment as well as clinical and behavioral (i.e. sexual mixing) input parameters (Dasbach *et al*. 2008 [23]). The UK model predicted a relative reduction of cervical cancer of 42.4%, 76.7%, 83.9% and 84.9% at 25, 50, 75 and 100 years, respectively; the US model predicted reductions of 62.4%, 79.1%, 83.0% and 83.6% at 25, 50, 75 and 100 years, respectively. These relative reductions coefficients were fairly comparable in a steady-state situation at 100 years (< 10% difference), although significant differences were present at 50 years. The US-based model was selected for use in the present analysis as it was calibrated for the extended range of HPV-related diseases (i.e. including HPV-related diseases other than cervical cancers and genital warts) at the time of the analysis.

### Sensitivity analyses

Sensitivity analyses were performed around cumulative vaccination coverage rate, ranging from 50% to 100% for both girls-only and girls and boys vaccination strategies (versus 70% in the base case) as well as compliance alternatively assumed to be ‘perfect’, e.g. 100% (while maintaining vaccine efficacy as its baseline value). Sensitivity analysis was also performed around duration of vaccine protection, in which a scenario of a shorter duration of protection equal to 32 years was assessed in line with the duration of protection assumed in a previously published analysis (Elbasha *et al*. 2010 [17]).

A final analysis was performed in which the girls-only vaccination program with 50% vaccine coverage was compared with the base case boys and girls vaccination program (with 70% vaccine coverage rate assumed). This comparative analysis was carried out to illustrate the potential impact of a higher coverage rate among boys than girls.

## Results

### Base case analysis

Results are presented for a steady state situation: at 100 years, when maximum vaccination effect is reached. Additional results at 50 years are provided in Table 4.

**Table 4:** Incremental benefit of a boys and girls vaccination strategy against HPV 6,11,16,18 vs. girls-only vaccination (results presented in a steady state situation, at 50 and 100 years; results from base case analysis)

**Gender**	**Disease**	**Annual number of HPV 6/11/16/18 cases**	**Annual number of cases avoided with girls only vaccination**		**Incremental number of cases avoided due to GNV (vs. girls only)**		**Relative reduction in remaining burden: GNV vs. girls only (%)**	
			**At 50 years**	**At 100 years**	**At 50 years**	**At 100 years**	**At 50 years**	**At 100 years**
Female	Genital warts	288,959	227,388	228,724	34,936	35,164	−56.7	−58.4
	Cervical cancer	23,254	13,848	19,728	958	1,362	−10.2	−38.6
	Vulvar cancer	2,702	873	2,286	67	157	−3.7	−37.8
	Vaginal cancer	1,146	406	981	31	66	−4.2	−39.9
	Anal cancer	2,929	821	2,330	80	258	−3.8	−43.0
	Head/neck cancer	2,531	701	2,020	67	220	−3.7	−43.0
	**Total cancers**	**32,562**	**16,649**	**27,345**	**1,203**	**2,062**	−**7.6**	−**39.5**
Male	Genital warts	325,722	202,671	202,587	85,740	87,900	−69.7	−71.4
	Penile cancers	1,091	93	197	156	542	−15.6	−60.6
	Anal cancers	1,699	313	1,067	180	402	−13.0	−63.6
	Head/neck cancers	12,707	2,555	8,203	1,449	2,967	−14.3	−65.9
	**Total cancers**	**15,497**	**2,961**	**9,467**	**1,784**	**3,911**	−**14.2**	−**64.9**
Female + Male	**Genital warts**	614,681	430,059	431,311	120,676	123,064	−65.4	−67.1
	**Total cancers**	48,059	19,610	36,812	2,987	5,973	−10.5	−53.1

GNV, gender-neutral vaccination (boys and girls vaccination); HPV, human papillomavirus.

The results of the base case analysis showed that in Europe, assuming a theoretical mean cumulative vaccination coverage rate of 70%, the introduction of a girls-only vaccination strategy was associated with a notable reduction in the incidence of HPV-related diseases in both males and females in comparison with screening alone (Table 4). With screening alone there were estimated to be 288,959 annual cases of genital warts and 32,562 cases of HPV-related cancer in females, with the corresponding figures in males being 325,722 and 15,497, respectively. Girls-only vaccination resulted in a 79% and 62% reduction in genital warts in females and males and an 84% and 61% reduction in female and male HPV-related cancers, respectively versus screening alone.

The benefits associated with the introduction of a boys and girls vaccination program were substantial, with the greatest benefits being reported in terms of the reduced incidence of genital warts. Vaccination of boys and girls led to additional 35,164 and 87,900 cases of genital warts being avoided in females and males, respectively (Table 4). Overall, vaccination of boys and girls was projected to lead to an 89% reduction in the incidence of genital warts in males and 91% reduction in females compared with a strategy of screening alone. Genital warts cases not prevented by girls-only vaccination are thus reduced by 58% for female cases and 71% for male cases due to extending vaccination to boys.

**Figure 1:**
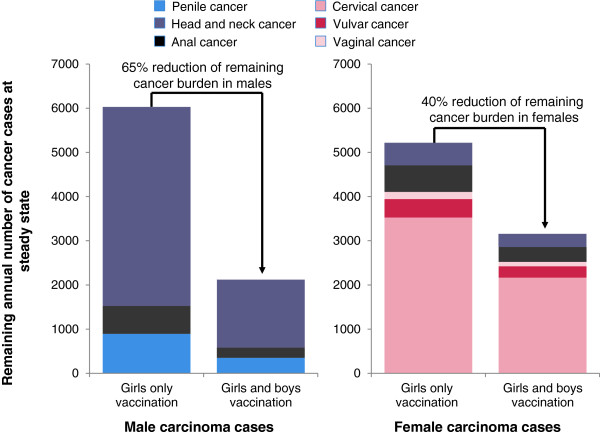
**Annual number HPV 16/18 related carcinoma cases among males and females when considering a vaccination strategy of boys and girls aged 12 versus girls only vaccination aged 12 (70% vaccine coverage rates assumed for all cohorts) - base case analysis presented at steady-state, 100 years.** The remaining annual burden of male HPV-related carcinomas is shown in the chart on the left side; remaining burden of female HPV-related carcinomas is shown in the chart on the right hand side.

The benefits of vaccination of boys and girls in terms of reducing the incidence of HPV-related carcinomas were also considerable. HPV-related cancers in males were reduced by 86% compared with screening alone (2,119 versus 15,497 cases). Extending vaccination to boys would therefore prevent an additional 3,911 male cases compared with girls-only vaccination (65% reduction) (Figure 1). The largest absolute incremental impact was observed for head and neck cancer where a reduction in the female and male absolute incidence from 5,015 to 1,828 cases was reported (an 88% reduction versus 67% with girls-only vaccination when compared with baseline screening alone).

Another substantial benefit of vaccination of girls and boys was in the incidence of male anal cancer; inclusion of boys in a HPV vaccination program led to an 86% reduction in the incidence of anal cancer (63% reduction with girls-only vaccination) in comparison with screening alone. Similarly, the vaccination of girls and boys was associated with a 68% reduction in the incidence of penile cancer, versus screening alone (compared with an 18% reduction with girls-only vaccination versus screening alone).

The vaccination of girls and boys would also lead to a benefit in terms of additional disease prevention among women as a consequence of indirect protection. In terms of the incidence of HPV-related cancer in women, in the base case analysis girls-only vaccination was associated with a reduction in HPV 16/18-related carcinomas of 84% versus screening alone; however, gender-neutral vaccination increased this figure to 90%. Overall, in females in the absence of vaccination there were 32,562 cases of HPV-related carcinomas, girls-only vaccination reduced this figure to 5,217 cases, but vaccination of girls and boys reduced the incidence of HPV-related carcinoma even further to 3,155 cases per year (Table 4).

Extending vaccination to boys and girls would therefore have the potential to reduce the HPV-related cancer burden in males by 65% versus girls-only vaccination. Due to indirect protection (herd immunity), vaccination of boys would allow a further reduction of female HPV-related cancer cases (2,062, 40%).

### Sensitivity analyses

**Figure 2:**
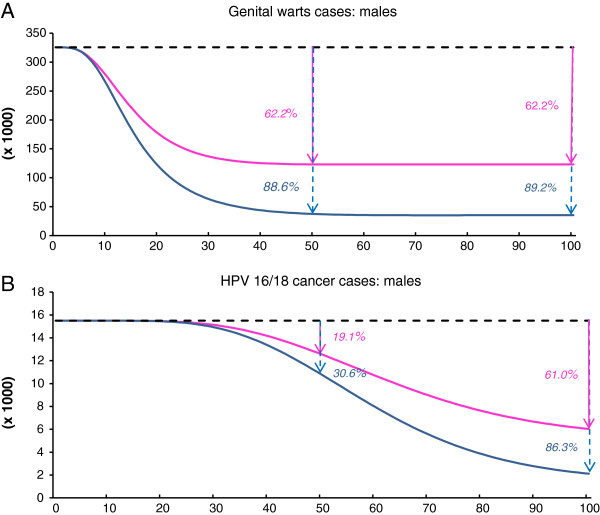
**Estimated annual remaining burden over the years 2012–2112 of HPV-related diseases when vaccinating 12-year old boys and girls versus girls only vaccination aged 12 (cumulative vaccination coverage rate 70%, lifetime duration of protection).** Remaining burden of HPV-related cases by subgroup of HPV conditions overtime under either girls-only vaccination or boys and girls vaccination. x-axis : years after implementation; y-axis: remaining number of cases. (**A**)-male genital warts; (**B**)- HPV 16/18 related male cancers. Black dotted-lines represent the base line (screening only). Pink lines represent the remaining cases in case of girls-only vaccination. Blue lines represent the remaining cases in case of boys and girls vaccination. Percents given are the relative reduction of incident cases compared with screening alone for a given year: either at 50 years or at 100 years. Over 50 years, (Area Under the Curve), vs. screening only were 32,788 HPV 16/18-related cancers cases and 7.0 million HPV6/11-related genital warts cases, respectively, which would have been avoided in males when vaccinating girls only. Additionally, 52,354 HPV 16/18-related cancers and 9.8 million HPV6/11-related genital warts cases would be avoided when vaccinating boys and girls.

**Figure 3:**
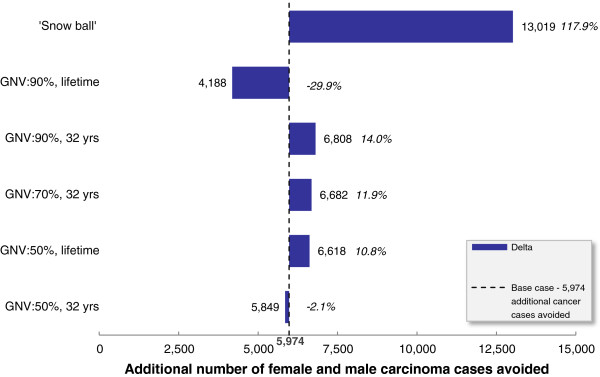
**Deterministic sensitivity analysis: impact of vaccinating boys and girls versus girls only vaccination**^**† **^**when considering the reduction of remaining burden of female and male carcinomas cases and percentage of relative variation versus base case analysis**^**‡.**^ GNV, gender-neutral vaccination (boys and girls vaccination). †: same coverage rate and duration of protection are assumed to be applied to boys and girls vaccination and girls only vaccination. ‡: boys and girls vaccination (cumulative vaccination coverage rate 70%, lifetime duration of protection).

Sensitivity analyses were restricted to parameters previously shown to be key drivers of epidemiological outcomes, i.e. vaccine coverage rates and the duration of protection (Elbasha, 2010 [17]). Varying vaccine coverage rates and duration of vaccine protection over time resulted in a notable variation in terms of the reduction in HPV-related disease burden (Table 5). In a scenario in which the vaccine coverage is 50% (scenario B) instead of 70% for boys and girls vaccination (while maintaining lifelong protection), 6,400 female and male carcinomas are not prevented (versus 70% coverage). Indeed, in scenario B fewer carcinomas cases would be avoided in comparison with the base case girls-only vaccination (Figure 2 and Figure 3). Conversely, increasing the coverage rate from 70% (base-case) to 90% (scenario C) would lead to an additional 3,453 carcinoma cases avoided across both genders. Sensitivity analysis also show that the waning effect (i.e. assuming 32-year long duration of protection instead of lifetime) has a considerable influence. Between 5,653 and 10,815 carcinoma cases (female and male) would not be prevented compared with base case boys and girls vaccination, depending on the level of coverage rate assumed (Table 5). The analysis also shows that the expected incremental benefit of vaccinating both boys and girls is the greatest in scenarios in which vaccine coverage rates in girls are low (Figure 3).

**Table 5:** Sensitivity analysis: number of cases avoided for the different boys and girls vaccination strategies versus boys and girls base case analysis (vaccine coverage rate: 70%, lifelong protection) and the corresponding relative reductions

**Absolute reduction (*increase*) of remaining cases (n) versus base case GNV vaccination strategy**							**Relative reduction (*increase*) of remaining cases (%) versus base case GNV vaccination strategy**				
**Duration of protection**		**Lifetime**		**32 years**							
**Coverage rate**		**50%**	**90%**	**70%**	**50%**	**90%**					
**Vaccination strategy**		**B**	**C**	**D**	**E**	**F**	**B**	**C**	**D**	**E**	**F**
Female	Genital warts	*40,271*	−24,568	*68,313*	*108,009*	*38,674*	*160.6*	−98.0	*272.5*	*430.8*	*154.3*
	Cervical cancer	*3,212*	−1,692	*5,685*	*8,853*	*3,092*	*148.4*	−78.2	*262.7*	*409.1*	*142.9*
	Vulvar cancer	*337*	−158	*568*	*925*	*285*	*130.3*	−61.2	*219.5*	*357.4*	*110.1*
	Vaginal cancer	*139*	−63	*236*	*388*	*116*	*140.3*	−63.7	*238.2*	*392.2*	*117.6*
	Anal cancer	*357*	−192	*598*	*978*	*300*	*104.6*	−56.3	*175.1*	*286.4*	*88.0*
	Head/neck cancer	*305*	−163	*504*	*832*	*250*	*104.6*	−55.9	*173.0*	*285.3*	*85.6*
	**Total cancers**	*4,351*	−2,269	*7,591*	*11,976*	*4,044*	*137.9*	−71.9	*240.6*	*379.6*	*128.2*
Male	Genital warts	*49,534*	−34,444	*77,279*	*122,846*	*43,104*	*140.6*	−97.8	*219.3*	*348.6*	*122.3*
	Penile cancers	*155*	−124	*237*	*358*	*130*	*44.1*	−35.2	*67.3*	*101.6*	*37.0*
	Anal cancers	*225*	−129	*351*	*576*	*173*	*98.0*	−56.0	*152.7*	*250.6*	*75.1*
	Head/neck cancer	*1,669*	−931	*2,636*	*4,331*	*1,306*	*108.6*	−60.6	*171.5*	*281.8*	*85.0*
	**Total cancers**	*2,050*	−1,184	*3,224*	*5,264*	*1,609*	*96.7*	−55.9	*152.2*	*248.5*	*75.9*
Female + Male	**Genital warts**	*89,805*	−59,013	*145,592*	*230,855*	*81,778*	*148.9*	−97.9	*241.4*	*382.8*	*135.6*
	**Total cancers**	*6,400*	−3,453	*10,815*	*17,240*	*5,653*	*121.4*	−65.5	*205.1*	*326.9*	*107.2*

HPV, human papillomavirus.

Negative values mean a reduction of the number of HPV burden of the disease and positive values mean an increase of the burden compared with base case boys and girls vaccination strategy (GNV).

In a scenario in which a vaccine efficacy of 100% was assumed (all other inputs were maintained at baseline values), a 69% decrease in male HPV-related carcinomas (minus 10,644 cases versus screening alone) would be achieved with a girls-only vaccination program, whereas a 61% reduction would occur in the base case girls-only vaccination (minus 9,467 cases versus screening only at steady state). When focusing on the impact of girls-only vaccination on cervical cancer, reductions of 85% and 86% were estimated in the base case and 100% vaccine efficacy scenarios, respectively. Extending vaccination to boys assuming a 100% vaccine efficacy would lead to a 96% reduction in the remaining burden in both male and female carcinomas (versus screening alone) compared with an 89% reduction assuming base case vaccine efficacy. This comparison confirms that vaccine efficacy related parameters estimates are key drivers of the results of modeling studies.

In a scenario that assumed 100% compliance (everything else being equal), girls-only vaccination would reduce cervical cancer burden by 91% (in comparison with 85% in the base case girls-only vaccination scenario) and extending vaccination to boys would lead to a 91% reduction of male HPV-related carcinomas (versus an 86% reduction in the base case girls-only vaccination scenario). A final sensitivity analysis aimed at exploring the leveraging effect of the introduction of HPV vaccination for boys on the vaccine uptake among girls was performed. It may be expected that vaccinating boys may increase the coverage rate among girls. When the base-case boys and girls vaccination (70% coverage) is compared with girls-only vaccination (50% coverage), such a “snow-ball” effect would prevent an estimated 13,019 carcinoma cases (half for each gender) and more than 160,000 cases of genital warts (Figure 3).

## Discussion

The results of the present analysis indicate that, assuming overall vaccination coverage of 70%, the vaccination of both girls and boys using the quadrivalent HPV vaccine was associated with notable incremental clinical benefits versus a strategy of girls-only vaccination. This is in contrast to other modeling studies, which concluded that the incremental impact of vaccinating boys was limited [18,19,21]. According to the present analysis, vaccination of boys and girls led to a 40% reduction in the incidence of HPV-related cancers and 58% reduction in the incidence of genital warts in females versus girls-only vaccination. Similarly, in males the incremental benefits associated with vaccination of boys and girls were a 65% reduction in the incidence of HPV-related carcinoma, including a 66% reduction in the incidence of HPV-related head and neck cancer and a 71% reduction in genital warts. Moreover, the incremental benefit of vaccinating both boys and girls was greatest in instances where the vaccination coverage rates in girls are sub-optimal, a finding that concurs with the findings from other modeling studies [18,19,21].

Differences in model structure and assumptions related to the natural history of HPV transmission and development of the disease, as well as differences in clinical outcomes, (used to assess the population-level clinical benefits) make direct comparisons between the outcomes of different models challenging. For example, Brisson *et al*. present their results in terms of HPV infection. They report the relative reduction in HPV-16/18 prevalence at equilibrium compared with no vaccination and the relative reduction in the incidence of vaccine-type infections over the first 70 years after the start of the vaccination, whereas our analysis reported the relative reduction of HPV-related diseases incidence rather than infections at equilibrium (100 years) [19]. Equilibrium was also assumed to be achieved at different time points across studies (from 50 years in Smith *et al*. to 100 years in the present study in line with previous work) [17,18]. Given the different approaches used in the current analysis and that of previously published analyses a detailed structural analysis and comparison of each model would identify the key differences in terms of underlying epidemiology, assumptions used and drivers of results. However, a detailed comparison of different available HPV models is beyond the scope of the present analysis.

Whilst previous modeling studies have focused on the reduction of the incidence of HPV infections and cervical cancer, data relating to the impact on vulvar, vaginal, penile, anal and head and neck cancer have until now been lacking. While girls-only vaccination would substantially reduce the incidence of HPV-related cancer in females and in some extent in males (due to herd immunity), vaccination of boys in addition to girls is associated with a substantial incremental benefit for both males (direct benefits) and females (indirect benefits). Indeed, in our base case, the estimated proportion of the maximum possible vaccine-conferred benefit to males (in terms of male HPV-related carcinomas) from gender-neutral vaccination, which would be achieved by a girls-only vaccination program, was as high as 71%. Nevertheless, this proportion may be reduced to 64% in scenarios assuming a lower vaccine coverage among girls (50%), and even further if a lower coverage rate occurred in conjunction with a waning effect (32 year long duration of protection). Such proportions refer to “proportional benefit achieved” as described by Smith *et al*., which primarily applied these calculations in terms of HPV-16 infection incidence data. The results suggest that in Europe, vaccination of boys and girls could prevent over 5,500 cases of HPV-related cancer annually (versus girls-only vaccination). Here, we present the number of HPV cancer cases avoided that are specifically due to HPV types 16 and 18. A previous analysis in the UK setting comparing quadrivalent and bivalent HPV vaccines assumed both vaccines provided some cross-protection against carcinomas caused by non-vaccine HPV subtypes. If vaccination does provide some cross protection for non-vaccine HPV types then potential clinical benefits estimated here may be conservative. However, research on cross-protection is currently ongoing and as such it was not included in this analysis [24].

With regard to the impact on genital warts, vaccination of boys and girls would reduce the incidence of genital warts by approximately 90%, which would likely lead to considerable economic benefits in terms of costs and resource use, indeed the economic burden of genital warts has been reported as being comparable to that of HPV related cancer and it has also been estimated that currently up 10% of visits to sexual health clinics are due to genital warts [14]. Additionally, the current analysis does not capture benefits in terms of quality of life or costs savings, which are also likely to be substantial.

Previous studies have shown that vaccine coverage in girls is a key driver of outcomes in both males and females [18,19]. Vaccine efficacy as well as compliance were also shown to be of particular interest when assessing the results. In Europe, vaccine coverage varies widely depending on setting due to differences in vaccination policy and modes of implementation (e.g. school based, invitation-based or available on request, and whether a catch-up program is in place for adolescent girls and young women). Countries with school-based vaccination programmes such as the UK have coverage rates of 80–90%, but school based programs only cover 13% of young women living in the EU. In settings where vaccination is administered on demand, such as France and Germany coverage rates are around 50%. In addition to substantial variations in vaccine coverage there are also marked differences across Europe with regard to uptake of cervical screening, which again is influenced by policies implemented on a national level. The proportion of women screened has been found to vary notably according to both age group and setting. For example, in Norway, Sweden and The Netherlands screening rates are high due to organized population-based programs, whereas in many other EU countries cervical screening remains opportunistic (e.g. France, Germany) with unequal access to screening and lower coverage or variation from one region to another (e.g. Spain, Italy) [25].

The results of the current analysis, together with the results of previous modeling analyses suggest that vaccination of boys and girls would be associated with the greatest benefit in settings where vaccine uptake among girls is low such as those countries that do not have a nationally coordinated vaccination program for females [18,26]. However, whether it is more feasible/more efficient to implement a strategy of vaccinating both boys and girls or increase vaccine uptake among girls only is an important policy decision that needs to be addressed on a national level given the variety of different vaccine implementation strategies (and hence coverage rates) in place across Europe. Indirect protection (herd immunity) in males is strongly dependent on vaccine coverage in females so the vaccination strategy used and coverage rate achieved is a key factor in determining the incremental benefit of the vaccination of boys at a national level. Additionally, ethical considerations are warranted regarding the type of vaccination program implemented (e.g. a consumer based approach versus a partially or fully subsided voluntary program versus compulsory vaccination).

The analysis presented here is associated with both strengths and limitations. Limitations of the current analysis include the fact that it does not consider the incidence of precancerous states such as cervical, vulvar, vaginal, anal or penile intraepithelial neoplasia, or capture temporal trends in HPV-related disease, such as the increasing incidence of head and neck cancer and anal cancer. Moreover, there is substantial uncertainty in the proportion of head and neck carcinoma attributable to HPV, which may be a contributing factor in the differences in the magnitude of clinical benefit reported across different studies. The present analysis was based on a proportion of 19% of head and neck cancers being attributable to HPV-16/18. This figure might be overestimated even if it is in line with estimates assumed in another recent modeling study by Smith *et al*. 2011 [18]. Given the magnitude of the burden of the disease of this subset of HPV-related conditions among males in particular, this is an area that potentially warrants further investigation.

Additionally, this analysis does not consider the quality of life benefit associated with the reduction in the incidence of HPV-related disease, which is also likely to be substantial. A further limitation of this analysis is that it is an exploratory analysis that presents mean findings relating to Europe as a whole and also that the model used here and applied to the European setting was based on a US-based dynamic transmission model with input data derived from the US setting, which may potentially limit its applicability to the European setting. In particular, the US-base case scenario (screening alone) is supposed to be consistent with what would be a European base case (screening alone). This is a strong underlying assumption given the specificities of screening implementation in the US and Europe in particular, in addition it is assumed that sexual behavior patterns and the age-structure of the population is similar between settings. Another limitation concerns the structure of the model in that it consists of a number of independent submodels (according to disease type), and incorporates the assumption that only subjects who are at risk of developing the disease can become persistently infected. As such, this means the transmission dynamics for female-only conditions (cervical, vaginal and vulvar cancer) are different from those where both males and females may be affected (head and neck and anal cancer) and from the male only penile cancer submodel. In addition, within Europe there are wide variations in vaccine uptake rates, screening coverage, HPV prevalence and transmission rates, and as shown here, vaccination uptake rates are a key driver of outcomes.

One of the key strengths of the analysis is that it assesses the benefits of male vaccination in all carcinomas that have an established causal link with HPV 6, 11, 16 and 18 whereas many previous analyses have focused primarily on the impact of vaccination in terms of cervical cancer incidence. The current analysis incorporates an extended number of HPV-related disease endpoints including subtypes of head and neck cancer and as such reflect the potential maximum clinical benefits that could be gained from different HPV vaccination scenarios (in comparison with a number of previous analyses that have focused largely on cervical cancer and genital warts only). It is also likely that the potential maximum clinical benefit reported in the current analysis would have been even greater if pre-cancerous states had been included in the analysis. Another strength is that this is the first analysis to present the potential public health impact at EU level of vaccinating boys and girls.

Further country-specific analyses that fully deal with uncertainty are required in order to guide policy decisions relating to the incremental benefits of vaccination of boys and girls. Until such data are available on a country by country basis, a pooled European-wide analysis may provide useful estimates, as well as serving as a valuable comparator for such analyses.

## Conclusions

This analysis is the first to assess the impact of gender-neutral vaccination in Europe; however, as noted country-specific analyses that take into account national vaccination policies, coverage rates and socio-ethical implications of different strategies may be required to assess the impact of gender-neutral vaccination at a national level.

These European-level results suggest that vaccination of boys and girls against HPV 6, 11, 16 and 18 would be associated with a marked incremental benefit in terms of a reduction in the incidence of HPV-related cancers and genital warts in males (31% to 77% and 30% to 99%, respectively) and would help decreasing the remaining burden of both HPV-related cancers and genital warts in females (14% to 68% and 21% to 98%, respectively). These figures represent the maximal potential benefit associated with vaccination due to the incorporation of an extended range of HPV-related cancers in the model, but may warrant updating in future analyses owing the current uncertainty that exists with regard to the proportion included cancers, in particular head and neck cancer that can be attributed directly to HPV.

Additionally, the incremental benefit is likely to be greatest in settings where vaccine coverage rates in females are not the highest. In addition to the clinical benefit, the notable reduction in the incidence of HPV-related disease is also likely to be associated with a substantial reduction in the economic burden associated with HPV-related cancers and genital warts.

## Competing interests

This study was supported by funding from Sanofi Pasteur MSD, Lyon, France. Xavier Bresse and Nathalie Largeron are employees of Sanofi Pasteur MSD. Rémi Marty and Stephane Roze are employees of HEVA, which has received consulting fees from SPMSD and Jayne Smith-Palmer is an employee of Ossian Health Economics and Communications, which has also received fees from SPMSD.

## Authors’ contributions

RM developed the Microsoft Excel 2003 impact model. XB, NL, RM and SR designed the study plan and XB and NL provided inputs data from the underlying transmission dynamic model. RM and XB performed the analyses. JSP wrote the manuscript. All authors read and approved the final manuscript.

## Grant support

This study was supported by funding from SPMSD SNC, Lyon, France.

## Pre-publication history

The pre-publication history for this paper can be accessed here:

http://www.biomedcentral.com/1471-2407/13/10/prepub

## Ackowledgments

Marjorie Adam, employee of Sanofi Pasteur MSD, for her critical review of the manuscript and support for its development. Ossian Health Economics and Communications, for their medical writing expertise and support in the production of this manuscript.
